# Prediction of patient's response to OnabotulinumtoxinA treatment for migraine

**DOI:** 10.1016/j.heliyon.2018.e01043

**Published:** 2019-02-28

**Authors:** Franklin Parrales Bravo, Alberto A. Del Barrio García, María Mercedes Gallego, Ana Beatriz Gago Veiga, Marina Ruiz, Angel Guerrero Peral, José L. Ayala

**Affiliations:** a Department of Computer Architecture and Automation, Complutense University of Madrid, Madrid 28040, Spain; b Carrera de Ingeniería en Sistemas Computacionales, Facultad Ciencias Matemáticas y Física, Universidad de Guayaquil, Guayaquil, Ecuador; c Neurology Department, “La Princesa” University Hospital, Calle de Diego Leon, 62, 28006 Madrid, Spain; d Headache Unit, Department of Neurology, Hospital Clínico Universitario de Valladolid, Valladolid, Spain; e CCS-Center for Computational Simulation, Campus de Montegancedo UPM, Boadilla del Monte 28660, Spain

**Keywords:** Computer science, Neurology, Bioinformatics, Medicine

## Abstract

Migraine affects the daily life of millions of people around the world. The most well-known disabling symptom associated with this illness is the intense headache. Nowadays, there are treatments that can diminish the level of pain. OnabotulinumtoxinA (BoNT-A) has become a very popular medication for treating migraine headaches in those cases in which other medication is not working, typically in chronic migraines. Currently, the positive response to Botox treatment is not clearly understood, yet understanding the mechanisms that determine the effectiveness of the treatment could help with the development of more effective treatments.

To solve this problem, this paper sets up a realistic scenario of electronic medical records of migraineurs under BoNT-A treatment where some clinical features from real patients are labeled by doctors. Medical registers have been preprocessed. A label encoding method based on simulated annealing has been proposed. Two methodologies for predicting the results of the first and the second infiltration of the BoNT-A based treatment are contempled. Firstly, a strategy based on the medical HIT6 metric is described, which achieves an accuracy over 91%. Secondly, when this value is not available, several classifiers and clustering methods have been performed in order to predict the reduction and adverse effects, obtaining an accuracy of 85%. Some clinical features as Greater occipital nerves (GON), chronic migraine time evolution and others have been detected as relevant features when examining the prediction models. The GON and the retroocular component have also been described as important features according to doctors.

## 1 Introduction

Migraine is a common neurological disorder characterized by recurrent headaches. Migraine attacks usually last for 4-72 h and involve moderate or severe intensity headaches which typically are worsened by routine physical activity, are of a pulsating nature, and are associated with nausea, vomiting, photophobia or phonophobia [1]. In clinical terms, migraine can be classified into two types according to the frequency of pain: episodic migraine (less frequent headaches) and chronic migraine. Chronic migraine is defined as a headache occurring on 15 or more days per month for more than 3 months, and which has the features of a migraine headache on at least 8 days per month [1]. Globally, approximately 2% of the population experiences chronic migraine [2]. In addition to the increased use of analgesic medication, visits to doctors, and visits to the emergency services, chronic migraine has a high socioeconomic cost, with higher direct and indirect costs. Furthermore, chronic migraine sufferers are more prone to anxiety, depression, other chronic diseases (respiratory, heart or circulatory) and more chronic pain, all of this associated with significant personal, societal, and economic burdens [3], [4].

The pharmacological treatment of chronic migraine is based on two pillars: abortive treatment of acute migraine attacks (that taken only in the acute pain phase) and preventive therapy. The latter is used to diminish the severity, frequency or duration of attacks. Preventive therapy includes additional benefits such as reduction of disability and enhancement of response to acute treatments [5]. It may also result in a reduction in health care costs [6].

Many classes of medication are used for migraine prevention: antiepileptic drugs, antidepressants, betablockers, calcium channel antagonists, serotonin antagonists, and botulinum neurotoxins, among others. In the case of chronic migraine, although all preventive treatments for migraine may be useful, only topiramate (a type of antiepileptic) and OnabotulinumtoxinA (BoNT-A) [7] have solid proven evidence for their use [8], [9], [10], [11], [12], [13]. BoNT-A has been an extended use treatment for chronic migraine since its approval in 2010 by the Food and Drug Administration in the United States (FDA), having also shown a more sustained effect and better tolerability than topiramate in the few comparative studies performed [14], [15]. BoNT-A can be injected under the skin (subcutaneous) or inside the muscles (intramuscular) in accordance with the so-called *The Phase III REsearch Evaluating Migraine Prophylaxis Therapy (PREEMPT)* paradigm. This injection method consists of using both fixed and follow-the-pain sites, with additional specific follow-the-pain sites considered depending on individual symptoms. This procedure should be carried out in repeated patterns after several months. Following the results of the initial clinical trials and subsequent published studies in real-life settings [16], [17], [18], [19], today it is known that 70-80% of patients with chronic migraine show an improvement with this treatment (improvement defined as a reduction in migraine attack frequency or days with attacks by at least 50% within 3 months, leading to a significantly improved functioning of the patients and their overall quality of life). Moreover, there is evidence that patients with chronic migraine who do not show the desired treatment response after the first cycle of BoNT-A treatment may indeed experience clinical improvement after one or two additional treatment cycles [20].

However, in clinical practice, about 20-30% of chronic migraineurs do not respond to BoNT-A. One of the most debated aspects in recent years has been the possible relationship between the clinical phenotype of migraine attacks and the response to BoNT-A. As has been mentioned in certain publications [21], it is very important to predict if the BoNT-A treatment will be effective in a patient. Knowing the phenotype-response relationship may help in the development of new treatments for the 20-30% of patients that do not respond to the treatment. Besides the cost, it would avoid the patients suffering the pain associated with the treatment.

In a real scenario of electronic medical records of migraineurs, we present a methodology for predicting whether or not the BoNT-A treatment will be efficient. Starting from the raw database provided by doctors, we preprocess it, identify the most promising feature to predict and then run several algorithms in order to get the prediction. Results show that it is possible to get an accuracy higher than 91% when employing the HIT6 [22] metric and 85% when this metric is missing. Moreover, our results show that some of the features leading to these accuracies are actually coherent with respect to the medical literature.

The rest of the paper is organized as follows. Section 2 describes the work related with some techniques applied to migraine and other illnesses. In Section 3, our methodology for predicting treatment results is explained. Section 4 describes the experiments and comparisons between different algorithms and our solution. Finally, our conclusions and future lines of work are presented in Section 5.

## 2 Related work

Several studies have looked at the clinical features of patients with migraine which may be associated with a favorable response to BoNT-A treatment, although conclusive results are not yet available for use in clinical practice. Possible predictors of a good response have been proposed: allodynia (painful hypersensitivity to superficial stimuli) [23], the unilateral character of a migraine [23], [24], associated migraine aura (visual, language, motor or sensory alterations occurring prior to pain) [25], or the build-up time to maximum pain (shorter time, better response to BoNT-A) [26]. Pain directionality also seems to be a possible clinical predictor. This feature refers to whether the headache feels like it is exploding, imploding or ocular. The term exploding refers to when the discomfort is felt pushing from the inside out. Patients suffering from imploding or ocular pain tend to be relieved with the BoNT-A treatment than those with the exploding [27]. Pagola et al. studied a number of possible clinical predictive features in parallel, including unilateral location of headache, pericranial muscular tension, directionality of pain, duration of migraine history and medication overuse, comparing responders to BoNT-A treatment with non-responders, but no significant differences emerged [28].

In order to find the most significant features of patients and classify them, there is a vast number of algorithms available [29]. C4.5, *k*-means, Support Vector Machines (SVM), Expectation-Maximization (EM) algorithm, PageRank, AdaBoost, *k*-NN, Naive Bayes, and CART are among the most common data mining algorithms used by the research community in many fields. A Feature Subset Selection (FSS) approach is typically applied first [30] in order to improve the accuracy of the classifiers. This approach has certain advantages, such as offering a better understanding of the prediction model or a better generalization by reducing *overfitting*. This problem happens when a prediction model is very closely adjusted to the training data, so it does not perform well when predicting new observations [31]. These methods have been applied to different neurological anomalies, for example: a feature extraction and selection from EEG signals in combination with a sleep stages classifier [32], an automatic seizure detection system for newborns [33], or to assess the feasibility of employing accelerometers to characterize the postural behavior of early Parkinson's disease subjects [34]. Furthermore, in order to improve migraine treatment predictions, we consider that simulated annealing (SA) [35] is a particularly interesting approach to take into account. SA is a stochastic, metaheuristic technique used in difficult optimization problems to approximate the global optimum of a given function in its search space. This approach has been widely employed to improve the performance of other algorithms. For example, SA has been used to improve FSS in [36]. Furthermore, SVM and SA have been combined to find the best selected features to increase the accuracy of anomaly intrusion detection in [37], and for a hepatitis diagnosis method in [38].

A key point to mention is how to measure the impact headaches have on daily life. In this sense, an important metric that allows the measurement of this issue is HIT6. The HIT6 [22] scale is a perceptional survey that is filled out by patients in order to measure their level of pain related with the migraine. In regular clinical practice, BoNT-A response is considered successful by doctors if it reduces migraine attack frequency or days with attacks by at least 50% within 3 months. Response features such as the HIT6 score (Headache Impact Test) are reflected less consistently. Thus, in our study, where data were obtained retrospectively through the review of clinical histories, we were able to obtain only a small set of patients for whom the HIT6 score had been collected. As a consequence, for the vast majority of the cases we must define an alternative way to determine the efficiency of the BoNT-A based treatment.

Therefore, although there is an ongoing research into the prediction of the appearance of migraines and even the effects of migraine treatment, to the best of our knowledge there is no existing method for predicting the efficiency of the BoNT-A treatment. For this purpose, we propose two methodologies that are customized for the migraine patients' clinical data, and which are able to deal with incomplete as well as heterogeneous data. Firstly, we present an approach that considers the medical HIT6 metric in order to predict the treatment success. Secondly, as this metric is rarely found in our medical databases, an alternative approach that uses SA in combination with classification and clustering methods is presented.

## 3 Methodology

**Figure 1:**
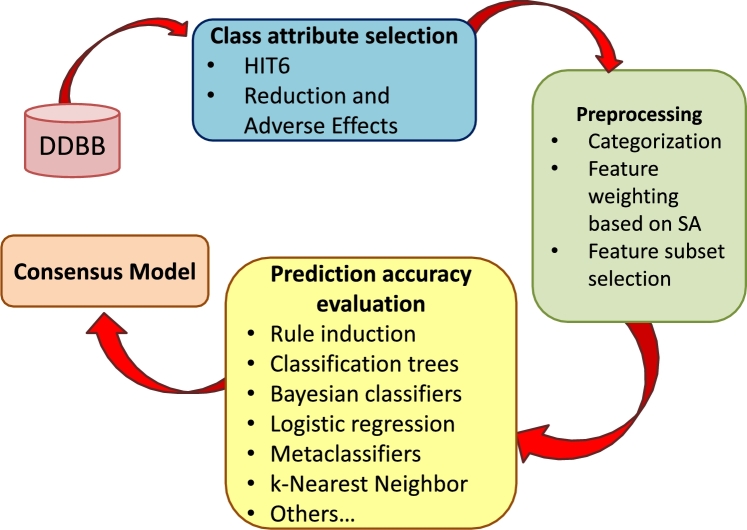
Framework diagram.

The issues involved in predicting the reduction of migraine symptoms when using the BoNT-A treatment will be described in this section. Figure 1 presents the framework on which this paper is based. Firstly, a database is loaded with the medical records from the two participating hospitals. Secondly, the class attribute is selected by considering the limitations of the medical records. Thirdly, clinical features are categorized in order to work with homogeneous data. Afterwards, a feature weighting mechanism based on simulated annealing or a FSS step is applied for improving the prediction accuracy. Finally, different classification algorithms are run and the best models are analyzed in order to detect clinical features that allow to predict the effectiveness of the treatment.

### 3.1 Clinical data

The data were collected retrospectively from the review of medical histories of patients with chronic migraine and under previous or current treatment with BoNT-A with follow-up at the Headache unit of two tertiary-level hospitals. To this end, the approval of the ethics committee of both hospitals was obtained under the documents ANA-TOX-2015-1 and PI-17-832 which are provided as supplementary content.

A total of 173 patients were included (116 from *Hospital Clínico Universitario* in Valladolid and 57 from *Hospital Universitario de La Princesa*, in Madrid). Sixty-two baseline features were categorized. It is necessary to mention that attributes, features, factors and variables are synonym terms in general. These features were related to the following points: clinical pain features, demographic features of patients, comorbidities, tested and concomitant preventive drugs, pain impact measures, and available analytical parameters. The latter were obtained from blood tests recorded in the clinical history which were performed for other reasons in the 3 months prior to, or 3 months after, the first infiltration, and included hemogram and liver, renal, thyroid, ferric, vitamin B12, folic acid and vitamin D profiles. The efficacy of BoNT-A was evaluated by comparing the baseline situation (before the first infiltration) and the situation after 12-16 weeks following each of the infiltrations, through the following parameters: number of days of pain per month, percentage reduction in days with pain, subjective intensity of pain, number of days of disability due to pain per month, HIT-6 scale score, drug consumption for pain and adverse effects of infiltration. Since this was a retrospective study, not all the data could be obtained for each patient in a systematic way.

Only 18 out of 173 records contained the perceptional HIT6 value before infiltrations, and only 12 and 3 contain this value after the first and second BoNT-A infiltrations, respectively. On the other hand, we found several efficiency indicators such as the reduction and adverse effects, which are provided in 102 and 86 registers for the first and second infiltrations, respectively.

To tackle classification and prediction for migraine treatment with BoNT-A, clinical data need to be previously processed in order to achieve a high level of accuracy. In fact, some patients are *non-respondent*, while others respond after the *i*th session. In order to predict the patients' behavior after the infiltrations, it is necessary to explore the patients' data before these take place. In other words, in order to predict the outcome after the *i*th session, the clinical data of the patient as well as the outcome after the (i−1)th infiltration are required. Nevertheless, some problems are encountered while evaluating these data. For example, a small set of patients with many features is typically present in our medical databases. In addition, the incompleteness of data is another problem that must be dealt with. Some features are given as continuous numeric values while other features are categorized by medics. All in all, it is hard to properly process all this information. As a consequence of these heterogeneous data, algorithms cannot infer a good model for predicting the outcome of the treatment. An example of these features can be observed in Table 1.

**Table 1:** Example of features in clinical data.

Toxin-age of onset (years)	Body mass index (kg/m^2^)	Hemoglobin (g/dL)	Creatinine (mg/dL)	Platelets (u/mcL)	Reduction effects (1-4)
51	20.39	13.4	0.71	213000	4
49	26.5	14.2	0.55	252000	2
36	23.15	13.5	0.44	304000	3
26	17.7	13.1	0.66	218000	2
31	NA	14.8	0.71	327000	1
50	NA	16.2	0.74	327000	3

### 3.2 Class attribute selection

In order to estimate the goodness of the solutions, it is necessary to define a metric, *class attribute*, that indicates how efficient the infiltration has been. In other words, class attribute is the selected clinical feature used to measure the effectiveness of treatment. According to doctors, some clinical features such as HIT6, effects reduction, adverse effects, or days with headache are good candidates for class attributes. The main problem is that the values of these features are not usually provided, with the exception of reduction and adverse effects. In this section, we first discuss the HIT6 value, which obtained a high level of accuracy in the experiments, as well as its limitations. In addition, a class attribute based on both the reduction and adverse effects is proposed to tackle the limitations imposed by the use of HIT6.

#### 3.2.1 HIT6

HIT6 is a highly specific perceptional value provided by doctors in order to measure the level of pain associated with migraine episodes. This value is obtained after patients fill out a standardized survey [22] consisting of six questions that capture the impact of headaches as well as their treatment. An example is shown in Table 2. These questions are:

1) When you have headaches, how often is the pain severe?
2) How often do headaches limit your ability to perform usual daily activities including housework, your job, homework, or social activities?
3) When you have a headache, how often do you wish you could lie down?
4) In the past 4 weeks, how often have you felt too tired to do work or daily activities because of your headaches?
5) In the past 4 weeks, how often have you felt fed up or irritated because of your headaches?
6) In the past 4 weeks, how often did headaches limit your ability to concentrate on work or daily activities?

**Table 2:** Hit6 Headache Impact Test example.

	never	rarely	sometimes	very often	always
Question 1	X				
Question 2		X			
Question 3			X		
Question 4				X	
Question 5					X
Question 6	X				

Points added	6+6=12	8	10	11	13

The values allowed for the answers are: never, rarely, sometimes, very often, and always. These values are graded with 6, 8, 10, 11 and 13 points, respectively. The HIT6 value is computed as the sum of all the individual scores. If the HIT6 value is 50 or higher, doctors interpret that the level of pain is enough to affect quality of life.

As this metric is perceptional, we have focused only on those database records containing the HIT6 value prior and after the infiltration. By defining the class attribute as the difference between the two values, as Equation (1) indicates, the bias due to different perceptions from different patients is diminished. According to [20], if the HIT6 value after the infiltration diminishes by more than 30%, the treatment is considered as successful, and unsuccessful otherwise. Hence, for this particular class attribute, only two categories have been defined, namely: successful and unsuccessful.

The HIT6 values are rarely found in clinical databases. In fact, only 12 patients from the clinical dataset from *Hospital Universitario de La Princesa* and *Hospital Clínico Universitario de Valladolid* had the HIT6 value before and after the first infiltration with BoNT-A. Therefore, although the achieved accuracy is high, as is shown in the experiments section, another class attribute must be defined to tackle other cases.

(1) $$HIT6_{dif}=HIT6_{b}-HIT6_{a}\;.$$

#### 3.2.2 Reduction and adverse effects

As a consequence of the HIT6 value being missing in many clinical records, the reduction (R) and the adverse (A) effects, which are more frequently found in the databases, have been selected to define the class attribute. Reduction and adverse effects are defined with values directly provided by doctors. These clinical features are quantified from 1 to 4, using 1 for the lowest and 4 for the highest level of effects.

*R* and *A* are measurable values from an objective point of view based on definitions. *R* is a clinical objective value categorized from 1 to 4 according to the percentage of reduction of days of migraine, being 1 when the percentage reduction of days of migraine is less than or equal to 25%, 2 for the interval between 25% and 49%, 3 for the interval between 50% and 74% and 4 when the percentage is greater than or equal to 75%. *A* is equal to 1 when there are no adverse effects, 2 when there are mild adverse effects (easily tolerated), 3 when there are moderate adverse effects (interfere with usual activities and may require suspension of treatment) and 4 when there are serious adverse effects (incapacitate or disable usual activities, and require suspension of treatment as well as medical intervention).

A high level of *R* indicates good treatment results, while high levels of *A* point to many adverse effects. Hence, in order to obtain a directly proportional feature, our class attribute ($N_{AC}$) has been determined by dividing *R* and *A*, as Equation (2) shows.

(2) $$N_{AC}=\frac{R}{A}\;.$$

In this work, a similar approach to the one based on HIT6 (two response categories: low and high) [20] has been considered for class attribute categorization, instead of the three categories (low, medium and high) used for the rest of the clinical features. In following this approach, two intervals (low and high) need to be defined before trying to predict the efficiency of the treatment when using $N_{AC}$ as class attribute.

Table 3 depicts an instance of the $N_{AC}$ computation using different values provided by the hospitals. Lower responses are labeled when the $N_{AC}$ value falls into the *(*$V_{min}$*, cut-off point)* interval, while high response labels are used for those values falling within the *(cut-off point,* $V_{max}$*)* interval. In this case, $V_{min}=0.25$ occurs when R=1 and A=4, while $V_{max}=4$ occurs when R=4 and A=1. We select a cut-off point of 1.40. The reason to use this value is the fact of trying to emulate the criterion used of the 30% decrease in the HIT6 value. It is considered as an effective response to the treatment in the PREEMPT clinical trial [20]. In this way, values lower than 1.40 represent the 30% of the values that $N_{AC}$ can take. Then, the low and high categories are defined with the intervals (0.25, 1.40) and (1.40, 4), respectively.

**Table 3:** Class attribute categorization.

Reduction effects (R)	Adverse effects (A)	R/A	Categorized value
1	1	1	low
2	1	2	high
3	2	1.5	high
1	2	0.5	low

### 3.3 Preprocessing

#### 3.3.1 Categorization of clinical features

In order to improve prediction accuracy for the BoNT-A treatment, the heterogeneous data from the hospitals is first categorized. The method selected for the categorization of our medical data is based on the mean and standard deviation. Applying this method makes it possible to work with more homogeneous values.

The mean and standard deviation categorization type centers the intervals around the mean (*μ*), and defines subsequent intervals by adding or subtracting the standard deviation (*σ*). For instance, if three categories are defined for a certain clinical feature, the intervals *(*$V_{min}$*,* μ−σ*)*, *(*μ−σ*,* μ+σ*)* and *(*μ+σ*,* $V_{max}$*)* are used to refer to value 1, value 2 and value 3, respectively. It should be noted that $V_{min}$ and $V_{max}$ are the minimum and maximum values of the data, respectively. By following a similar strategy it is possible to define multiple intervals. The pseudocode of the intervals generation for a feature categorization is presented in Algorithm 1.

**Algorithm 1:**
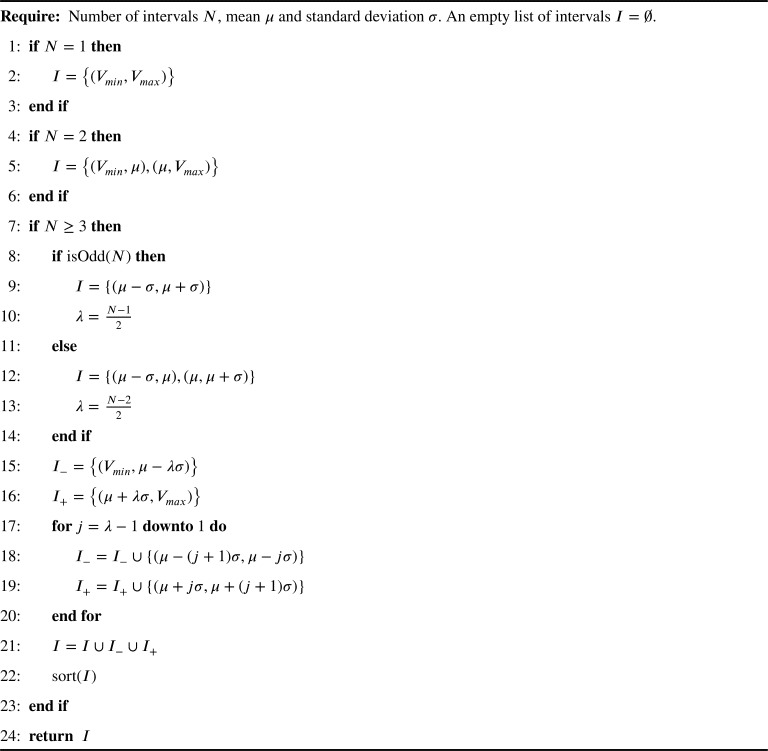
Intervals for categorizing features

#### 3.3.2 Feature subset selection (FSS)

This technique makes it possible to enhance the prediction efficiency of the classification and clustering methods, as it just considers the most influential features when predicting the class attribute value. This approach has certain advantages, such as offering a better understanding of the prediction model and a better generalization by reducing overfitting [39]. Several approaches have been designed to implement the FSS technique as the filter, wrapper or embedded method [40]. The filter type method selects features without considering the model. In this approach, the emphasis is placed on the general features such as the existent correlation with the class to predict. The wrapper method tries to find interactions between features by evaluating subsets of them. Finally, the embedded method considers certain search algorithms in order to combine the advantages of the first two methods.

Different FSS algorithms [30] have been applied in order to determine the most relevant clinical features when obtaining the treatment response prediction. Table 4 shows the main features of the four studied FSS implementations, namely: feature evaluator and search method. C4.5 is the classifier selected to work together with the WrapperSubsetEval and ClassifierSubsetEval methods to measure the worthiness of the subset of features within the dataset.

**Table 4:** Description of FSS methods used in experiments.

FSS type	Feature evaluator	Description	Search method
FSS1	CfsSubsetEval	Evaluates the worth of a subset of features by considering the individual predictive ability of each feature along with the degree of redundancy between them.	BestFirst
FSS2	WrapperSubsetEval	Evaluates feature sets by using a learning scheme.	BestFirst
FSS3	ChiSquaredAttributeEval	Evaluates the worth of a feature by computing the value of the chi-squared statistic with respect to the class.	Ranker
FSS4	ClassifierSubsetEval	Evaluates feature subsets on training data or a separate hold out testing set.	BestFirst

#### 3.3.3 Weighting features

In order to enhance the accuracy of the classification and the clustering algorithms, the simulated annealing method (SA) [35] has also been considered as a preprocessing step. SA is a randomized search method for optimization. Our purpose is to find those weights that allow us to do improvements in the representation of the numeric labels encoded by doctors for each infiltration.

When applying this method to our problem, we define the error (100% - accuracy) as the objective value to be diminished. In this way, the SA algorithm will be able to optimize a weighted sum of features. The approach has been implemented using the Hero library [41]. This library implements the “Natural Optimization” proposed by De Vicente et al. [42], which means that the temperature does not need to be given because it is continuously tuned while running the SA algorithm (Equation (3)). In addition, an initial random weight vector solution (one weight per attribute) will be given as input to the SA algorithm. After this, the error rate will be computed and saved as the initial fitness value to be minimized. Then, a mutation over one of the weights will be performed. The procedure will be repeated until completing a defined maximum number of iterations (*N*).

**Figure 2:**
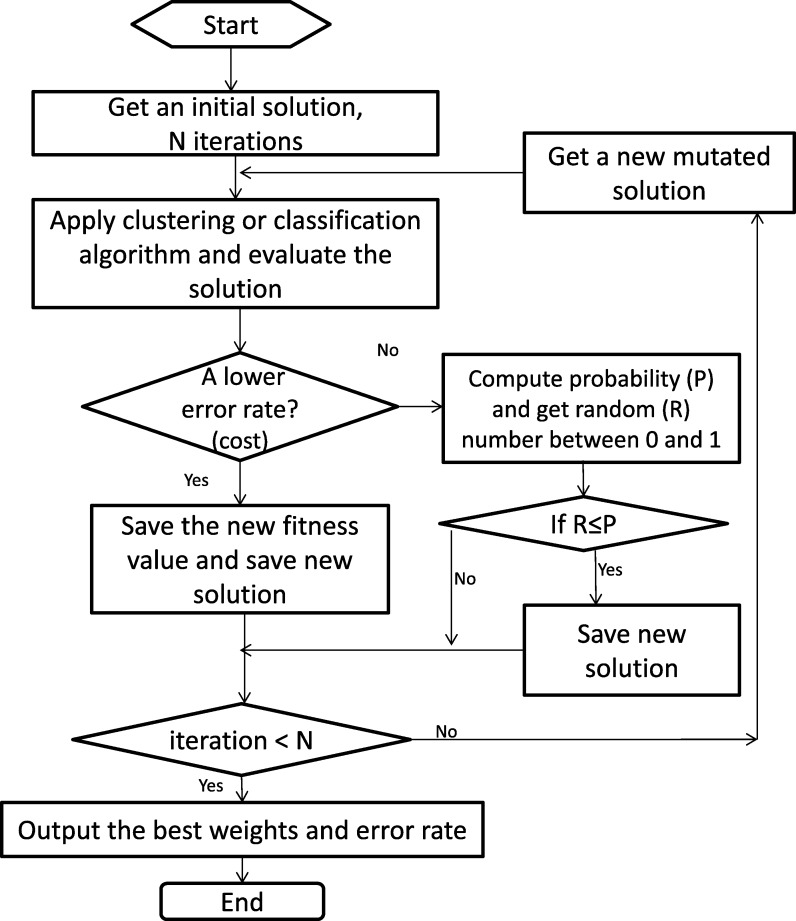
Flowchart with the proposed Simulated Annealing-based methodology.

Figure 2 depicts a flowchart with the methodology, where *T* is defined by Equation (3)

(3) $$T=\frac{K\times (C_{min}-C_{init})}{N}\;,$$

where *N* is the number of iterations, *K* is a constant that refers to the backward degree and time/quality trade-off and has been set to 1, and $C_{min}$ and $C_{init}$ refer to the current minimal cost and initial cost, respectively. The energy difference is defined in Equation (4).

(4) $$E_{diff}=C_{sol}-C_{min}\;,$$

where $C_{sol}$ is the cost of the solution. Finally, the probability (*P*) to compare with the random number (*R*) is given by Equation (5). *P* is the probability of changing to a new solution. This is calculated when accuracy is not lower than the fitness value. When R≤P, SA moves the solution to another point within the search space to avoid being trapped in a local minimum.

(5) $$P=e^{\left(-E_{diff}/T\right)}\;.$$

### 3.4 Prediction accuracy evaluation

#### 3.4.1 Classification

The problem of prediction could be tackled by using classification algorithms which identify categories for new records based on the previous data (training dataset) [39]. These records (observations) have been previously labeled by doctors (supervised learning). This implies that given N records characterized by given prediction features, the training data will be transformed into a classification model able to predict the label of the class attribute for every new record with some level of success (accuracy).

In our particular case, the $N_{AC}$ class attribute is used whenever the HIT6 value is not available (for the majority of the patients). Several state-of-the-art classifiers [29] (e.g. TAN, RIPPER, C4.5 or NB tree algorithms) are applied in order to compare their prediction accuracy and to gain a general idea of possible ways to improve the results. All these algorithms and its parameters are described in Table 5.

**Table 5:** Descriptions of classifiers used in experiments.

Classification algorithm	Description	Parameters
Naive Bayes	Numeric estimator precision values are chosen based on analysis of the training data.	No parameters
IBk	*k*-nearest neighbors classifier.	*k*-NN=2, Linear Search algorithm
RIPPER	Propositional rule learner, Repeated Incremental Pruning to Produce Error Reduction.	Pruning=true, Seed=1
C4.5	Generates a pruned or unpruned C4.5 decision tree.	Confidence factor=0.25, Seed=1
Logistic	Builds and uses a multinomial logistic regression model with a ridge estimator.	maxIts=-1, Ridge=1 ⋅ 10^−8^
AdaBoostM1	Meta classifier: Boosts a nominal class classifier.	Classifier=Decision Stump, Iterations=10, Seed=1
Bagging	Meta classifier: Bagging a classifier to reduce variance.	bagSizePercent=100, Classifier=Random tree or C4.5, Iterations=10, Seed=1
LMT	Builds classification trees with logistic regression functions at the leaves.	minNumInstances=15, numBoostingIterations=-1
NBTree	Generates a decision tree using Naive Bayes classifiers for the leaves.	No parameters
Random forest	Builds a forest of random trees.	Number of trees=100, Seed=1
Random tree	Builds a tree considering K randomly chosen features for each node. Performs no pruning.	minNum=1, Seed=1
REPTree	Builds a regression(decision) tree using information gain and variance and prunes it using reduced-error pruning.	maxDepth=-1, minNum=2
DecisionStump	Builds a tree that make predictions based on the value of just a single input feature (also called 1-rules).	No parameters
SVM	Builds a model that assigns new examples to one category or the other, making it a non-probabilistic binary linear classifier.	cacheSize=40, cost=1, kernelType=radial

#### 3.4.2 Clustering

This technique works by grouping all the records or observations into different groups called “clusters”, each of them containing elements with similar features [39]. In our study, we have considered two different clusters (low and high) to indicate the result of the treatment. In this technique, we consider $N_{AC}$ as the class attribute. Different state-of-the-art clustering algorithms such as *k*-means, expectation- maximization (*EM*) and farthest-first have been selected to predict treatment response. The clustering algorithm is usually an unsupervised method. However, the values of the class attribute have been tagged. Additionally, two clusters have been defined to categorize responses to treatment, so we will use the cluster algorithms as supervised clustering. In this sense, the clustering algorithm is applied to classified examples and has the objective of identifying clusters that have a high probability density with respect to a single class. As mentioned by Eick et al. (2005) [43], the fitness functions used for supervised clustering are significantly different from the fitness functions used by traditional clustering algorithms. The fitness function evaluates a clustering based on the number of clusters and class impurity. The impurity refers to measure the percentage of minority examples in the different clusters of a determined cluster.

In addition, a majority voting metacluster composed of the three aforementioned algorithms has also been considered. Hence, the result of the metacluster will be the dominant value among the three clustering algorithms. An example of this behavior is presented in Table 6.

**Table 6:** Metacluster behavior.

EM	*k*-means	Farthest-First	Predicted value
low	high	low	low
high	low	high	high
low	high	high	high
low	low	high	low

### 3.5 Consensus model

As was mentioned in Section 1, the pathophysiological features that determine the positive or negative response to the migraine treatment are not known yet [44]. We can take advantage of the use of a consensus model to reveal these features. The idea is not to build a consensus predictor model, but to understand the most relevant clinical features that exist in the majority of the induced prediction models of the best classifier.

Ensemble techniques can help us analyze feature relations with the construction of consensus models to make new and relevant findings [45], [46]. In this sense, Armañanzas et al. [47] have proposed an ensemble interaction network for unveiling biological relations when analyzing Alzheimer's disease. In that study, many Bayesian *k*-dependence models are induced to output a gene interaction network composed of arcs (edges). An occurrence threshold *t* is defined to output the most frequent edges above a predefined confidence level (the 0.999 quantile is used in order to retain just the most important connections). The list of interaction networks and the associated list of highly relevant features are obtained to reveal or corroborate biological hypotheses in this disease. Other studies [48], [49] can be found in the literature with similar purposes.

In this paper, this technique is applied in order to group different prediction models (decision trees) produced by the best classifier in terms of accuracy for both infiltrations. This is done with the purpose of finding explicit features and relations between medical features that influence the treatment response prediction. In the FSS method (Section 3.3.2), these features are selected before the construction of the prediction model by using different metrics. In the ensemble interaction network, the idea is to invert the feature selection process of FSS, which means that the relevant features will be selected after, and not before, the construction of the prediction models.

We define the decision tree model as the graph G(V,E), where *V* represents the vertex list (features as vertices) of the model and *E* represents the list of edges (relations between vertices) of the model. The interactions in the decision tree consist of parent-child edge relations. Nodes are filled with the feature values and edges represent the parent-child relation from the decision tree model. Edges for the first level of the induced models will have a null value as vertex *u* in the edge tuple (u,v) because the roots of decision trees do not have parents. Many decision trees will be induced by a resampling method (*k*-fold cross validation) together with the SA optimization. For each level of the decision tree, the most frequent clinical features will be taken into account. After this, an interaction network will be depicted with edges whose frequencies are higher than a reliability threshold *t*. Edges occurring more than *t* times for each level of the tree will be retained. After that, these relevant features will be contrasted with the most important features obtained with the FSS methodology.

The threshold value *t* will be different for each level of the tree. Edges will be sorted according to their frequency of appearance in a given level. In order to retain only one vertex as root of the consensus decision tree, we will retrieve only the 0.99 quantile (*t* value) for the first level of edges of the induced models. For the rest of the levels, the 0.9 quantile will determine the *t* value for retaining the most important edges. These quantile values have been defined by considering the 0.999 quantile applied by Armañanzas et al. [47] but modified with the purpose of retaining only one root and multiple important child nodes in the consensus decision tree proposed. All these steps are summarized in Algorithm 2. Table 7 presents the functions and definition of variables used in the algorithm.

**Algorithm 2:**
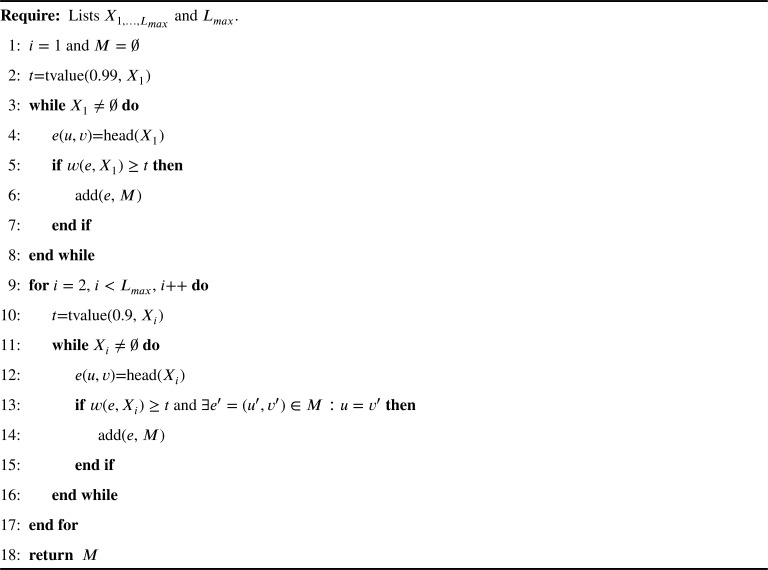
Relevant features in consensus trees

**Table 7:** Description of variables and functions employed in Algorithm 2.

Name	Description
*v*	Vertex or node.
*e*(*u*,*v*)	Edge *u* → *v*, where *u* is parent of *v*.
*w*(*e*,*X*_*i*_)	Weight of an edge *e*. *w*(*e*,*X*_*i*_)=|{*e* ∈ *X*_*i*_}|.
*X*_*i*_	The edges list at level *i* of the induced prediction models (decision trees) for a given infiltration.
*M*	List of nodes that conform the consensus tree.
*L*_*max*_	A defined maximum number of levels to explore for the consensus tree construction.
tvalue(*q*, *X*)	Calculates the *t* value given the quantile (*q*) value and the *X* list.
head(*X*)	Returns and removes the first element of the *X* list.
add(*e*,X)	Adds *e* to the *X* list.

## 4 Experimental

In order to test the proposals, our framework was implemented with Java and using the Hero library [41] as well as Weka 3.8 [50]. As was mentioned in Section 3.1, the clinical dataset consists of 102 patients that have undergone the first infiltration and 86 the second infiltration during BoNT-A treatment. These records are divided into two datasets for each infiltration, one for training-test and another for validation. For the first infiltration, 76 and 26 records were employed for the training-testing and the validation datasets, respectively. In the second infiltration, 64 and 22 records were used for the training-testing and the validation sets, respectively. When training-testing the predictions over the class attribute, the *k*-fold cross validation (*k*=10) was applied without the use of a validation set [51]. The results presented in this section are based on the measured accuracy of the *k*-fold cross validation. The *k*-fold cross validation method has been used to avoid reporting overoptimistic results of classifier algorithms because of overfitting. The validation set was used to verify the results found by the *k*-fold cross validation process. Moreover, the *k*-fold cross validation results were used (as fitness value) to improve the SA parameter tuning process (feature weighting) in the experiment presented in Section 4.2.3. Clinical data were provided by the *Hospital Clínico Universitario* in Valladolid, and the *Hospital Universitario de La Princesa* in Madrid, both being in Spain.

### 4.1 HIT6 prediction

In this experiment, only the clinical data belonging to patients whose database record contains the HIT6 value were considered. The purpose is to predict high-low differences in HIT6 values before and after infiltrations, as was explained in Section 3.2.1. The HIT6 value is required before and after the first infiltration with BoNT-A in order to apply Equation (1). Only 12 patients meet this requirement. In this initial test, the feature subset selection (FSS) step was not considered. Moreover, the validation dataset was not taken into account because of the number of records.

Due to the small size of the dataset with the HIT6 value, an exhaustive cross-validation method called Leave-One-Out Cross-Validation (LOOCV) is applied for training-testing the classifier algorithms of this section. This method has been applied with the purpose of creating all possible partitions of *n* cases, when the cardinality of a training set is fixed to n−1 and the cardinality of a testing set is 1.

Table 8 presents the accuracy values after employing the class attribute defined by Equation (1) in combination with several state-of-the-art classification algorithms. As can be observed, some algorithms show a high level of accuracy. For example, the AdaBoost, DecisionStump, C4.5 and NB tree algorithms possess an accuracy that is higher than 91%. These algorithms are based on rules or trees, with the exception of the AdaBoost meta classifier algorithm, which boosts a nominal class classifier (DecisionStump).

**Table 8:** HIT6-difference prediction.

Classification algorithm	First infiltration		
	Accuracy	Sensitivity	Specificity
Naive Bayes	66.66%	0.00	0.72
IBk	66.66%	0.00	0.72
RIPPER	75.00%	0.50	0.80
C4.5	**91.66%**	1.00	0.90
Logistic	50.00%	0.20	0.71
AdaBoostM1 (DecisionStump)	**91.66%**	1.00	0.90
Bagging (Random tree)	75.00%	0.50	0.80
Bagging (C4.5)	66.66%	0.00	0.72
LMT	58.33%	0.25	0.75
NBTree	**91.66%**	1.00	0.90
Random forest	75.00%	0.00	0.75
Random tree	66.66%	0.33	0.78
REPTree	75.00%	0.00	0.75
DecisionStump	**91.66%**	1.00	0.90
SVM	75.00%	0.00	0.75

Means	74.44%	0.39	0.79

Medians	75.00%	0.25	0.75

The values of sensitivity and specificity are also presented in Table 8. These values are considered because they are more important than high accuracy values in many medical problems [52]. The sensitivity measures the fraction of positive cases that are classified as positive, while the specificity measures the fraction of negative cases classified as negative. In our case, the positive values will be the patients who have a good therapeutic response (labeled as “high”) to the treatment, while the negative cases will be the ones that obtain a bad response (labeled as “low”). Overall, the classifiers that obtain high accuracies (greater than 90%) also have high values of specificity and sensitivity. This means that the number of false positives and false negatives is very low.

Despite the positive results, there are very few patients possessing HIT6 values for the first infiltration (12 records). As it has been mentioned above, it is not usual to have this information in our clinical databases. In fact, predictions on the second infiltration have not been performed because only 3 patients registered their HIT6 value after this infiltration. As a consequence of this reduced amount of records, we can only conclude that HIT6 seems to be a good choice as a measurement of the treatment effectiveness. Therefore, we can only recommend the collection of such clinical feature in the medical records of migraine treatment and more specifically in the treatment with BoNT-A. Future research may show that HIT6 is a good severity index for measuring the effectiveness in the migraine treatment. Consequently, other strategies for treatment classification need to be analyzed.

### 4.2 Reduction and adverse-effects-based prediction

Because of the lack of availability of HIT6 values, in this section the new class attribute defined by Equation (2) is considered. In the same fashion as defined in Section 3.2.2, the reduction-adverse effect values are used to measure the accuracy comparison among different classification and clustering algorithms of this section.

#### 4.2.1 Classification methods

Several classifiers were applied in order to select the best algorithm in terms of accuracy. In Table 9, the accuracy percentage of different classifiers is presented. High values of sensitivity and specificity are also presented to visualize the correct prediction of high and low responses to treatment. Some algorithms achieved an accuracy of more than 60% for the two class values classification (high-low) on the first infiltration treatment prediction. By considering a probability function that predicts the two class values with a probability of 50% for each one, it can be observed that these algorithms do not achieve high accuracies. Moreover, 63.72% of class values in the complete dataset take the value of “high” as treatment response after the first infiltration. Therefore, classifying all instances as “high” can ensure an accuracy of 63.72% (baseline accuracy). Similar results were achieved in the second infiltration response prediction, where the “low” response represents 52.32% of all the dataset. These low accuracies may be a consequence of the large number of features in comparison with the reduced number of records in the medical data (52 and 56 features vs 102 and 86 records for first and second infiltrations, respectively). Additionally, sensitivity and specificity values are less than or equal to 0.65. The exception is the IBk classifier for the first infiltration, whose sensitivity value is 0.78, which involves an excellent detection of patients who respond positively to treatment.

**Table 9:** Accuracy percentage of some classic classification methods.

Classification algorithm	First infiltration			Second infiltration		
	Accuracy	Sensitivity	Specificity	Accuracy	Sensitivity	Specificity
Naive Bayes	57.89%	0.65	0.44	51.56%	0.50	0.53
IBk	50%	0.78	0.35	59.37%	0.61	0.58
RIPPER	56.57%	0.61	0.33	59.37%	0.60	0.59
C4.5	50%	0.65	0.22	48.43%	0.46	0.50
Logistic	51.31%	0.61	0.37	51.56%	0.50	0.53
AdaBoostM1 (DecisionStump)	53.94%	0.60	0.28	45.31%	0.41	0.48
Bagging (Random tree)	50%	0.57	0.09	54.68%	0.54	0.55
Bagging (C4.5)	43.42%	0.54	0.15	57.81%	0.58	0.57
LMT	**63.15%**	0.63	1.00	56.25%	0.56	0.56
NBTree	51.31%	0.60	0.33	**62.50%**	0.61	0.64
Random forest	55.26%	0.60	0.27	51.56%	0.50	0.53
Random tree	50%	0.58	0.30	56.25%	0.55	0.58
REPTree	57.89%	0.61	0.29	48.43%	0.44	0.50
DecisionStump	59.21%	0.61	0.25	51.56%	0.50	0.52
SVM	61.84%	0.62	0.00	50.00%	0.48	0.51

Means	54.12%	0.62	0.31	53.64%	0.52	0.54

Medians	53.94%	0.61	0.29	51.56%	0.50	0.53

#### 4.2.2 Feature subset selection

In Table 10, Table 11, the clinical features selected by methods of Table 4 are presented for the first and the second infiltrations, respectively.

**Table 10:** FSS on first infiltration training data.

Features selected	FSS1	FSS2	FSS3	FSS4
Onset age of toxin treatment	X			
Retroocular component	X		X	
Migraine chronic	X		X	
**Calcium antagonists**	X	X	X	X
**Enolism**	X	X	X	X
Vitamin B12		X		X
First grade family with migraine		X		X

**Table 11:** FSS on second infiltration training data.

Features selected	FSS1	FSS2	FSS3	FSS4
Retroocular component	X		X	
**GON**	X	X	X	X
Pneumopathy	X		X	
Dermopathy	X		X	
Vitamin B12	X		X	
1-Red. and Adv.Eff.clasif	X		X	

For the first infiltration, calcium antagonists and enolism features were selected by the four evaluated FSS methods. For the second infiltration, only the previous greater occipital nerve block (GON) was taken into account by the four evaluated FSS methods. In addition to these, two features were selected in the first and the second infiltrations: the retroocular component and vitamin B12.

In the experiment of this section, only the features presented in the Table 10, Table 11 have been taken into account for building the prediction models of the first and second infiltration respectively. Table 12 presents the accuracy of classifiers when just using these features. A noticeable improvement in the second infiltration response prediction was achieved when using this approach. More specifically, the Naive Bayes algorithm achieved an accuracy of 70.31% in contrast to the 62.50% obtained by the NBTree classifier without applying the FSS method. Moreover, the sensitivity value of this classifier for the second infiltration is equal to 0.77, which involves an excellent detection of patients who respond positively to the treatment for such infiltration.

**Table 12:** Accuracy percentage of classifiers with feature subset selection.

Classification algorithm	First infiltration			Second infiltration		
	Accuracy	Sensitivity	Specificity	Accuracy	Sensitivity	Specificity
Naive Bayes	**64.47%**	0.67	0.56	**70.31%**	0.77	0.66
IBk	53.94%	0.60	0.31	42.18%	0.42	0.42
RIPPER	51.31%	0.58	0.17	68.75%	0.69	0.69
C4.5	57.89%	0.61	0.33	60.93%	0.60	0.62
Logistic	65.78%	0.68	0.59	62.50%	0.64	0.62
AdaBoostM1 (DecisionStump)	59.21%	0.62	0.40	62.50%	0.62	0.63
Bagging (Random tree)	56.58%	0.62	0.38	64.06%	0.67	0.63
Bagging (C4.5)	60.52%	0.63	0.46	54.68%	0.54	0.56
LMT	63.15%	0.65	0.54	54.68%	0.55	0.54
NBTree	55.26%	0.59	0.14	59.37%	0.59	0.60
Random forest	56.57%	0.62	0.38	65.62%	0.68	0.64
Random tree	52.63%	0.59	0.29	59.37%	0.60	0.59
REPTree	59.21%	0.62	0.38	65.62%	0.68	0.64
DecisionStump	63.15%	0.63	0.67	56.25%	0.58	0.56
SVM	64.47%	0.65	0.63	67.18%	0.67	0.67

Means	58.94%	0.62	0.42	60.93%	0.62	0.60

Medians	59.21%	0.62	0.38	62.50%	0.61	0.62

Despite this promising improvement, response predictions for the first infiltration were not significantly improved when comparing the baseline accuracy of 63.72% explained in Section 4.2.1. Furthermore, an accuracy of 70% for two class prediction is not close to the 91% accuracy obtained when using HIT6, as shown in Section 4.1. In addition, the sensitivity and specificity values are less than or equal to 0.67, which implies that false positive and false negatives are appearing with certain frequency.

#### 4.2.3 Feature weighting with SA

As was mentioned in Section 3.3.3, SA is applied with the purpose of improving the representation of the numeric labels encoded by doctors (preprocessing). The number of iterations was defined as two million. Table 13 presents the accuracy of the classifier algorithms when applied together with the SA technique. Their accuracies improved significantly when using this technique. The best accuracy was achieved with random tree (≈85% and ≈86% for the first and the second infiltrations, respectively). The relevant medical factors found by the effective combination of SA and random tree are presented in Section 4.2.5. The sets of the best features found by FFS and the SA process are compared and discussed in Section 4.2.5 and 4.3.

**Table 13:** Accuracy percentage of classifiers with simulated annealing.

Classification algorithm	First infiltration			Second infiltration		
	Accuracy	Sensitivity	Specificity	Accuracy	Sensitivity	Specificity
Naive Bayes	64.98%	0.61	0.25	67.64%	0.63	0.68
IBk	75.00%	0.80	0.67	81.25%	0.83	0.80
RIPPER	67.11%	0.66	0.75	72.02%	0.72	0.75
C4.5	61.24%	0.70	0.50	73.44%	0.68	0.81
Logistic	67.11%	0.75	0.56	62.50%	0.61	0.65
AdaBoostM1 (DecisionStump)	65.79%	0.67	0.67	64.07%	0.68	0.62
Bagging (Random tree)	75.00%	0.73	0.85	81.25%	0.83	0.80
Bagging (C4.5)	61.85%	0.61	0.36	73.43%	0.75	0.72
LMT	65.79%	0.64	1.00	67.62%	0.70	0.68
NBTree	60.53%	0.64	0.47	67.62%	0.70	0.68
Random forest	80.77%	0.79	0.85	81.25%	0.83	0.80
Random tree	**84.61**%	0.85	0.83	**85.94**%	0.82	0.90
REPTree	67.11%	0.67	0.70	63.63%	0.64	0.63
DecisionStump	65.79%	0.64	1.00	67.62%	0.69	0.68
SVM	75.00%	0.73	0.85	81.25%	0.83	0.80

Means	69.18%	0.70	0.69	72.70%	0.73	0.73

Medians	67.11%	0.67	0.70	72.02%	0.70	0.72

Regarding the sensitivity and specificity, we can observe that some classifiers such as IBk, bagging with random tree, random forest, random tree and SVM possess values greater than 0.80. Of special consideration is the random tree algorithm, which also achieves a high accuracy. Given their high sensitivity and specificity values we can conclude that these classifiers perform a good detection of positive and negative responses to treatment in both infiltrations.

On the basis of the results, we can observe that non-deterministic classifier algorithms (random tree and random forest) combined with SA perform the best in Table 13 (an accuracy higher than 80%). Previous results (Table 8, Table 9, Table 12) show that the best classifiers were deterministic. Then we can conclude that SA becomes an important factor, as it helps to optimize non-deterministic algorithms. Looking for the lowest fitness, SA moves the solution within the search space to avoid being caught in a local minimum in non-convex problems [53], and this benefits the non-deterministic algorithms.

Looking more closely at the results of sensitivity and specificity values of Table 9, Table 12, we can see an overall improvement in the specificity of nearly all classification methods because of the SA pre-processing. In general, those now correctly classified cases are female patients with chronic migraine without aura, no retroocular component, nausea and vomiting, less than 48 months of migraine time evolution, previous radiofrequency treatment, topiramate and at least two other preventives drugs tested before toxin and calcium antagonists.

**Figure 3:**
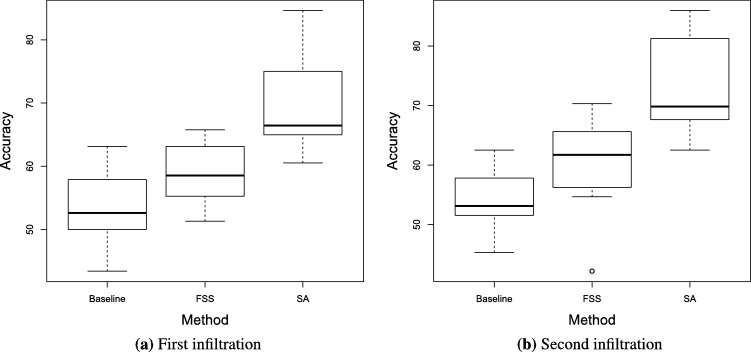
Accuracies distribution for first and second infiltration under the methods used in Table 9, Table 12, Table 13.

With the purpose of statistically validating if the improvement in classification due to the FSS and SA methods is significant, the Kruskal-Wallis (non-parametric) test with two degrees of freedom was carried out between the accuracy values of Table 9, Table 12, Table 13 for both infiltrations. This test gave us the results of $p=1.753\cdot 10^{-6}$ for the first infiltration and $p=2.146\cdot 10^{-6}$ for the second infiltration. These values, being less than 0.05, guarantee us that there is a significant difference in the distributions of values among groups. The distribution of classification accuracy obtained under the baseline (classifiers without any improvement), FSS and SA methods used in Table 9, Table 12, Table 13 for both infiltrations are presented in Figure 3. Table 14 shows the results of the Nemenyi post-hoc test for detecting which pairs of methods are significantly different. According to this test, the classifiers improved with SA had a highly significant difference (p<0.01) in comparison to baseline classifiers and when considering FSS. On the contrary, FSS-baseline difference is not significant (p>0.05).

**Table 14:** Nemenyi post-hoc test for accuracies of Table 9, Table 12, Table 13.

Pair-methods comparison	First infiltration		Second infiltration	
	Mean rank difference	*p*	Mean rank difference	*p*
Baseline-FSS	−8.50000	0.1588	−10.17857	0.0720
SA-FSS	15.03571	0.0034	13.39286	0.0108
SA-Baseline	23.53571	1.2 ⋅ 10^−6^	23.57143	1.1 ⋅ 10^−6^

#### 4.2.4 Clustering methods

In this section, several clustering methods were evaluated by considering the class attribute defined by Equation (2) in combination with the SA algorithm. Cluster methods use heuristic criteria that seek to group patient records that contain the maximum closeness between them (Section 3.4.2). The number of clusters was established as two. This number was decided in order to cover the two values that take the class attribute to predict (high-low). The number of iterations that were executed to optimize the weights of features by SA was established in one million. As in the previous tests, clinical data belonging to the first and second infiltrations were taken into account. Table 15 shows the accuracy percentages for the four clustering methods described in Section 3.4.2. The farthest-first clustering method achieves the highest accuracy (88.47%) for the first infiltration. This prediction is better than the one obtained when using the random tree and SA combination for the same infiltration. Nevertheless, in general we have observed that this method does not obtain a big accuracy difference as random tree and SA combination. Supervised clustering may have achieved better accuracies than traditional clustering for the first infiltration, because the dataset has a high probability density with respect to a single class in that infiltration [43].

**Table 15:** Accuracies of clustering algorithms when using simulated annealing.

Algorithm	First infiltration	Second infiltration
Meta-Cluster	80.77%	**81.82**%
EM	73.08%	77.28%
*k*-means	65.38%	**81.82**%
Farthest-First	**88.47**%	63.64%

#### 4.2.5 Consensus model

Section 3.5 discusses the importance of studying a consensus model with the prediction models built for the first and the second infiltration of the treatment. With our medical datasets, the random tree and SA combination has proved to be the best classifier for both infiltrations. We have induced many random tree models instead of clustering algorithms or random forest. This decision was taken because the models generated by clustering methods and by random forest are difficult to interpret in terms of relevant features. Moreover, only the most frequent features for each level of the studied models were taken into account. An important point to emphasize is that the ensemble tree obtained is not intended to be a prediction model of the treatment response for each infiltration. On the contrary, this allows us to know the most frequent clinical features and the relations that appear in the majority of the prediction models selected (only prediction models with the highest accuracies).

Many random trees were induced by the resampling method (using *k*-fold cross validation with *k*=10) with the SA optimization (used for the experiments in Section 4.2.3). These relevant features are contrasted with the important features obtained when using the FSS methodology in Section 4.2.2. The prediction models selected for induction were the models that achieved an accuracy of 84.61% and 85.94% for the first and the second infiltrations, respectively. 5000 prediction models for each infiltration were generated from 50 SA weighted feature vectors, which makes it possible to achieve the highest accuracies for both infiltrations. Regarding the root vertex of the ensemble tree, the 0.99 quantile was applied as the *t* value. Taking into account Table 16, this value was equal to 1449.08 for the first infiltration (t = 1552.56 for the second infiltration). In this way, GPT was selected as the root of the consensus tree for the first infiltration because of its high frequency (1690 times). In a similar way, *t* was defined as the 0.9 quantile from the empirical edge frequency distribution of the other levels of the ensemble tree for both infiltrations.

**Table 16:** Frequency of clinical features for the first level (root) of random trees on the first infiltration.

Feature	Frequency
GPT	1690
Hemoglobin	1056
Emergency days by month	668
Migraine days by month	516
History of migraine status	500
Vitamin B12	482
Creatinine	464
HTA	350
Platelets	310
Onset age of toxin treatment	300
Serum iron	300
Calcium antagonists	300
Headache days by month	248
Gastropathy	248
Radiofrequency Treatment	230
Urea	230
Enolism	222
GOT	184
GGT	182
Analgesics abuse	176
Retroocular component	168
Catamenial	120
Neuromodulator	114
Unilateral pain	112
Triptan days by month	110
Local painful pressure of greater occipital nerve (GON)	100
Chronic migraine	94
Nausea(Vomiting)	92
Folic acid	90
Tricyclic antidepressants	70
Migraine type	54
First grade family with migraine	50
Oral Preventive Treatment	42
Betablockers (B-blocker)	42
Concomitant antihypertensive treatment	28
Alkaline phosphatase	18
Migraine evolution time	18
Analgesic days by month	12
Symptomatic treatment	10

**Figure 4:**
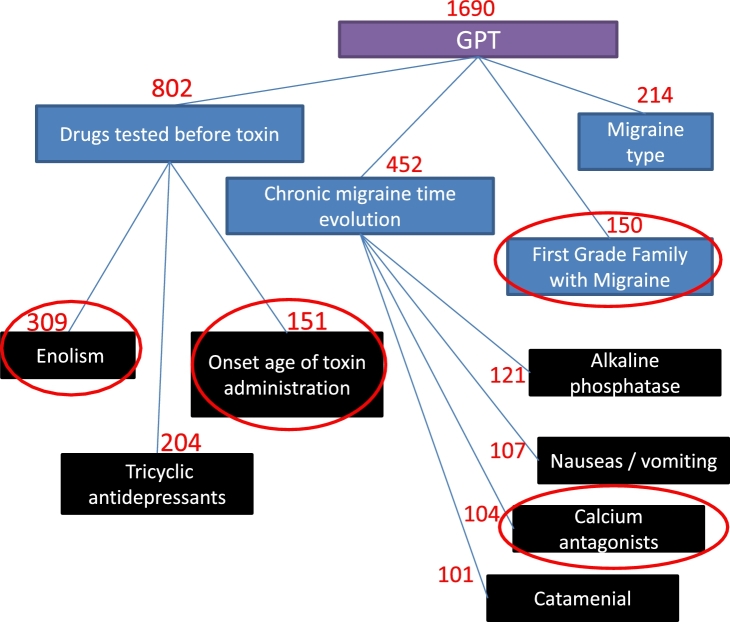
Consensus tree using random tree models for the first infiltration.

**Figure 5:**
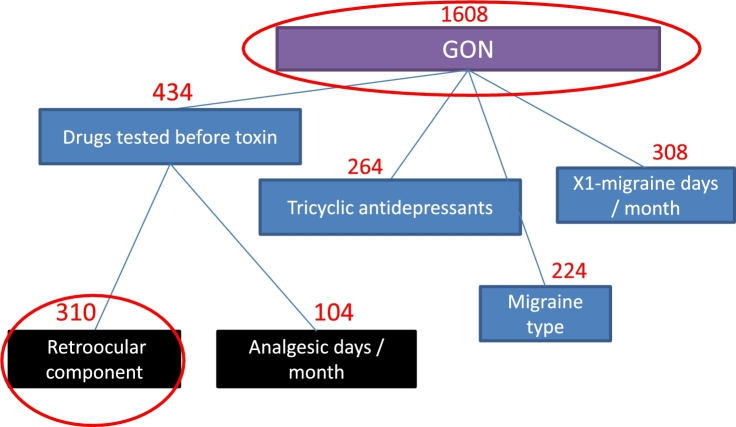
Consensus tree using random tree models for the second infiltration.

Figures 4 and 5 present the most frequent clinical features for both infiltrations. An important aspect to note is that we have defined the $L_{max}$ value as 3 for both infiltrations. This value was established by considering the comprehension of the resultant consensus tree as a primordial criterion. Higher values of this parameter would allow us to see more features, but comprehension could decrease when contrasting these features with those obtained with the FSS method. In this sense, a consensus tree with a low number of leaves is more understandable. Features were filled with different box colors that indicate different levels of the tree (purple, blue and black for levels 1, 2 and 3 of the tree). In addition to this, red circles indicate the features that were selected when performing the FSS methods presented in Table 10, Table 11 for the first and the second infiltrations, respectively. With this analysis, the sum of the frequency of edges will not necessarily be equal to the frequency of their parent nodes because not all edges are represented in the consensus tree, but only the edges that exceeded the *t* value.

According to these consensus trees, the most important factors that influence the prediction of the treatment response to BoNT-A for the first infiltration are GPT, drugs tested before toxin, migraine type, chronic migraine time evolution, first grade family with migraine and others. For the second infiltration, the factors are GON, drugs tested before toxin, x1-migraine days by month, tricyclic antidepressants, retroocular component and analgesic days by month. Although it is true that in the experiments of the previous sections it has been shown that SA obtains a better accuracy than the use of FSS, we can point out that they select similar features with respect to the selected by FSS. For example: first grade family with migraine, enolism, onset of toxin administration and calcium antagonists were indicated by both methods as relevant features for the first infiltration while for the second were the GON and the retroocular component.

### 4.3 Medical discussion

The transformation of episodic migraine into chronic migraine occurs over months or years and involves atypical pain modulation and central sensitization triggered by repetitive inputs from sensitized peripheral sensory neurons [54]. The exact analgesic mechanism of action of BoNT-A is only partially known. The main hypothesis is that the toxin exerts its antinociceptive action inhibiting peripheral sensitization. BoNT-A lowers neuropeptide and neurotransmitter release from peripheral sensory neurons, thereby indirectly reducing central sensitization, the hallmark of chronic migraine [55], [56].

The aforementioned data suggest that the pharmacological response to BoNT-A might be better when the migraine headache is “trigeminal” in pain location and corresponds to reflex trigeminal-autonomic activation [56], [57]. As a consequence, BoNT-A action may be more effective in migraineurs who overactivate peripheral trigeminal endings during the attack, and such patients may be identified by means of easily obtainable patient-reported clinical findings, such as pain location or direction (unilateral, implosive-retroocular), the presence of cranial autonomic symptoms (allodynia) and cortical spreading depression signs (aura) [56]. Other data such as the response to anesthetic block of the greater occipital nerve (GON) or its local painful pressure (positive palpation) might suggest the same. Many authors believe that a therapy which blocks peripheral transmission of pain signals from extracranial areas prior to central sensitization will successfully disrupt migraine headache propagation [25], [58], [59].

In our results, the GON and the retroocular component were also selected as relevant features when building our most accurate prediction models. Therefore, we can conclude that the relevant features extracted by FSS and the consensus random trees are coherent with respect to the medical literature.

## 5 Conclusions

This study assesses the application of data mining techniques to the prediction of BoNT-A treatment efficiency for migraine patients. In this work, two methodologies are presented. The first is based on the perceptional HIT6 value, which is not frequently found in our clinical databases. In order to overcome this limitation, a second methodology based on more widely available clinical features is presented. A preprocessing strategy based on simulated annealing is proposed to select the best way to represent the information in terms of prediction accuracy. The combination of simulated annealing and the random tree algorithm allows us to obtain an accuracy of 85% without considering the rarely found HIT6 value.

In addition, relevant clinical features extracted when using FSS and consensus random trees have been presented. Features such as GON and the retroocular component have also been described as important clinical features to consider for migraine treatment in the medical literature. This knowledge allows us to conclude that the features considered in our prediction models are coherent with respect to the medical literature.

In the future, the use of bootstrapping-based techniques to obtain a predictive model from the random sampling generated will be contempled. In addition, some optimizations need to be done in order to increase the prediction accuracy.

## Declarations

### Author contribution statement

Franklin P. Bravo, Alberto A. Del Barrio García, José L. Ayala: Conceived and designed the experiments; Performed the experiments; Analyzed and interpreted the data; Wrote the paper.

María M. Gallego, Ana B. Gago Veiga, Marina Ruiz, Angel G. Peral: Analyzed and interpreted the data; Contributed reagents, materials, analysis tools or data.

### Funding statement

This work was funded by the Instituto Carlos III Healthcare Research Fund (Pl15/01976) and the Ministry of Education, Science, Technology and Innovation (SENESCYT) of the Government of the Republic of Ecuador (8905-AR5G-2016). The project was co-financed by the European Regional Development Fund.

### Competing interest statement

The authors declare no conflict of interest.

### Additional information

No additional information is available for this paper.
